# Bioinformatics Genes and Pathway Analysis for Chronic Neuropathic Pain after Spinal Cord Injury

**DOI:** 10.1155/2017/6423021

**Published:** 2017-10-15

**Authors:** Guan Zhang, Ping Yang

**Affiliations:** ^1^Department of Neurobiology, Chongqing Key Laboratory of Neurobiology, Third Military Medical University, Chongqing 400038, China; ^2^Cadet Brigade, Third Military Medical University, Chongqing 400038, China

## Abstract

It is well known spinal cord injury (SCI) can cause chronic neuropathic pain (NP); however its underlying molecular mechanisms remain elusive. This study aimed to disclose differentially expressed genes (DEGs) and activated signaling pathways in association with SCI induced chronic NP, in order to identify its diagnostic and therapeutic targets. Microarray dataset GSE5296 has been downloaded from Gene Expression Omnibus (GEO) database. Significant analysis of microarray (SAM), Kyoto Encyclopedia of Genes and Genomes (KEGG) pathway enrichment analysis, and pathway network analysis have been used to compare changes of DEGs and signaling pathways between the SCI and sham-injury group. As a result, DEGs analysis showed there were 592 DEGs with significantly altered expression; among them Ccl3 expression showed the highest upregulation which implicated its association with SCI induced chronic NP. Moreover, KEGG analysis found 209 pathways changed significantly; among them the most significantly activated one is MAPK signaling pathway, which is in line with KEGG analysis results. Our results show Ccl3 is highly associated with SCI induced chronic NP; as the exosomes with Ccl3 can be easily and efficiently detected in peripheral blood, Ccl3 may serve as a potential prognostic target for the diagnosis and treatment of SCI induced chronic NP.

## 1. Introduction

Neuropathic pain (NP) is a common consequence following spinal cord injury (SCI), which compromises a person's life satisfaction and quality. It is estimated that the mean cost was $47,518 for each SCI patient with NP in the USA [1]. According to the statistics of the GoPubMed website (http://www.gopubmed.org/web/gopubmed/), the molecular mechanisms of NP following SCI remain elusive. NP in SCI can be classified as “at-level” pain [2–4], “below-level” pain [2, 3], and “above-level” pain [5–7]. According to the International Spinal Cord Injury Pain Classification system [8], pain experienced at or within three dermatomes below the neurologic level of injury is considered at-level neuropathic pain, while pain that is present more than three dermatomes below the level of injury is classified as below-level neuropathic pain. While at-level pain results from lesion of nerve roots and/or the spinal cord and is felt at the corresponding segment, below-level pain is a central pain caused by damage to spinal cord pathways, suggesting different pathological mechanisms of pain generation [9, 10]. However, there is not too much description for above-level neuropathic pain following SCI in previous study. Not rarely, patients suffer both at-level and below-level pain, but at-level pain seems to appear earlier than below-level pain clinically [11].

The prevalence of NP syndromes in the general population is as high as 7 to 8% [12, 13], and approximately 30–50% of patients with a SCI will develop chronic NP [14–16]. And, according to previous studies [17], a total of 140 participants were analyzed, 70 of them were SCI-NP subjects and the remaining 70 controls did not show neuropathic symptoms. SCI can result from trauma, tumor, infection, and degenerative condition; among them the traumatic SCI plays a pivotal role in inducing chronic NP [18]. There are annually 0.25–0.5 million SCI cases around the world and more than 90% are due to traumatic injury [19]. SCI results in cell loss, disruption of neural circuitry, and chronic functional impairment [20]; thus patients with chronic NP after SCI may benefit from strategies aiming to promote neurogenesis, neural plasticity, and functional recovery such as human umbilical cord-derived mesenchymal stem cell transplantation [21], astrocyte transplantation [22–25], and neural stem cell transplantation [26, 27]. The mechanisms underlying SCI induced chronic NP remain elusive, and recent advance in neuroscience has implicated that SCI is a polygenic disease and its pathogenic mechanism is associated with changes of gene expression; therefore identification of related genes in chronic NP after SCI could provide new insights into gene function as well as potential diagnostic and therapeutic targets. In this study, we used microarray technology to identify differentially expressed genes (DEGs) and activated signaling pathways in association with SCI induced chronic NP in a mouse SCI model.

## 2. Material and Methods

### 2.1. Data Source

Microarray technology is a widely used high-throughput tool for measuring gene expression [28–30]. Moreover, previous studies have shown that data from DNA microarray analysis can be reliable and useful for identifying novel targets for clinical diagnostic and therapeutic approaches [31]. Thus, we used the microarray expression profiles (GSE5296), which were extracted from the GEO (https://www.ncbi.nlm.nih.gov/geo/) database, to identify DEGS associated with SCI induced chronic NP. A C57BL6 mouse SCI model was used (https://www.ncbi.nlm.nih.gov/geo/query/acc.cgi?acc=GSE5296). The experimental group (*n* = 12) was subjected to a moderate injury at the T8 spinal cord segment under isoflurane anesthesia. Total RNA was extracted from sections of rostral regions, caudal regions, and lesion centre from T8 spinal cord injury (0.4 cm in length each one). The expression of genes was detected at a series of time points: 0.5, 4, 24, 72 h, and 7 and 28 days after injury, respectively. According to clinical circumstance, patients commonly experience NP during the initial 3–6 months and 3–5 years after SCI [2]. Therefore, it must be noted that neuropathic pain did not appear during the time points we studied. The control group is sham-injured (*n* = 8), with laminectomy only. Global changes were evaluated using Affymetrix Mouse Genome 430 2.0 arrays. The experiments were performed three times.

Altered DEGs and signal pathways were compared between injury group and sham-injury group in different regions: rostral, lesion centre, and caudal regions and at serial time points. The lesion centre has the most significant alteration in DEGs and signal pathways, and thus the dates in different time point from lesion centre were chosen to perform the next analysis before normalization processing by RMA (robust multichip average).

### 2.2. Data Preprocessing

The RMA method [32] is for computing an expression measurement with three steps' process: background-correction, normalization, and summary. The method includes a probe-specific background-correction and a probe selection strategy in which a subset of probes with highly correlated intensities across multiple samples is chosen to summarize gene expression.

### 2.3. Analysis Methods

GCBI platform (https://www.gcbi.com.cn/gclib/html/index) was mainly used in the whole process. Initially, DEGs were significantly identified in the spinal cord total RNA samples from C57BL6 mouse model of contusion injury in comparison with samples from animals of laminectomy only. Significant analysis of microarray (SAM) is used to study DEGs. *P* = 0.05 was used as the significance threshold of screening DEGs, and fold change > 2 was used as the threshold to determine the significance of gene expression difference. Cluster analysis based on Pearson correlation calculation was used to ensure that the screened genes perfectly expressed the differences between SCI group and sham-injury group. Furthermore, GO functional [33] and KEGG analysis were performed to identify the altered pathway involved in the SCI. Significantly enriched GO terms and KEGG pathways with Fold Discovery Rate (FDR) < 0.05 and *P* < 0.05 were screened out. Finally, pathway net analysis was to explore the relationships among each pathway. What is more, it must be noted that SAM (significant analysis of microarray) is used to study DEGs (differentially expressed genes), while RMA (robust multichip average) is for normalization procession, and their function are totally different.

## 3. Results

### 3.1. Data Preprocessing from Lesion Centre

**Figure 1:**
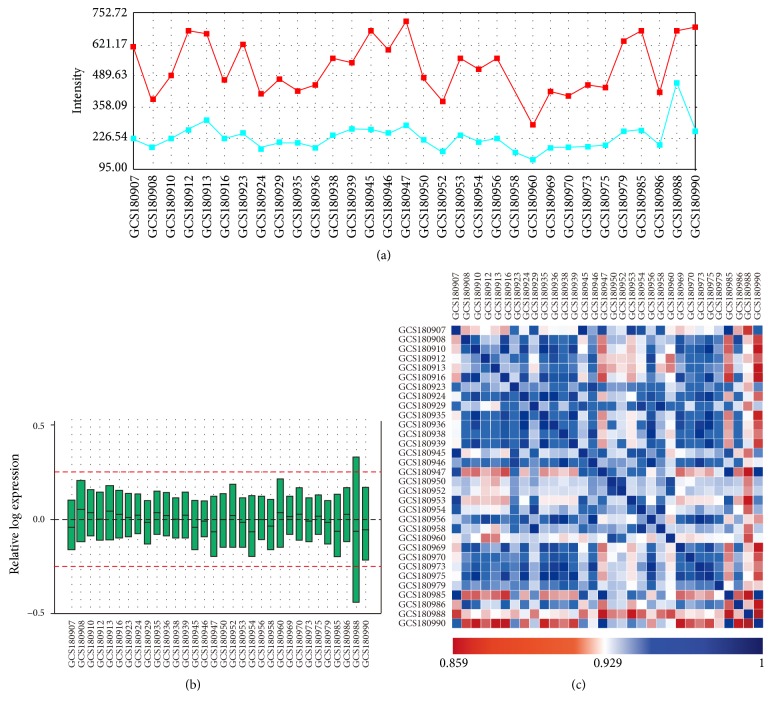
The background and signal value for each sample. Red represents the average of the values of the sample signals for each sample. Blue represents the average of the background values for each sample. It demonstrated that, after excluding the influence of background, the signal values of the samples are still high (a). The horizontal axis represents the name of samples, while the vertical axis represents the expression value after log conversion. The black lines stand for median, which can be used to identify the degree of standardization after normalization of all samples by the package of R/Bioconductor. It can be seen that the black lines were almost on the same line (b). Sample correlation calculated by injury associated genes expression. Both the horizontal axis and the vertical axis represent the name of samples. The gene expression level from different sample was calculated with Pearson correlation. The closer the point is to the blue color, the greater the correlation is between the two samples. It shows that the correlations of all samples are basically very strong (c).

The original data were preprocessed by RMA function with the Affymetrix package of R language [34]. The original CEL files were switched into probe expression measures, and the probe-level data were converted into gene names by an annotation package supported by the GCBI platform. After excluding the influence of background, the signal values of the samples are still high (Figure 1(a)), assuring the reliability of the analysis results. It can be seen that the black lines were almost on the same line (Figure 1(b)), indicating an excellent degree of standardization, which ensure the accuracy of subsequent data processing. The correlations of all samples are basically very strong (Figure 1(c)), providing the basis of the subsequent cross analysis and system analysis. The data preprocessing results showed that the samples were sufficiently, precisely, and stably enough to support the following analysis.

### 3.2. Screening of Differentially Expressed Genes from T8 Lesion Centre (Ccl3 Was the Maximum Changed Gene Expression Profile among the 592 DEGs)

**Figure 2:**
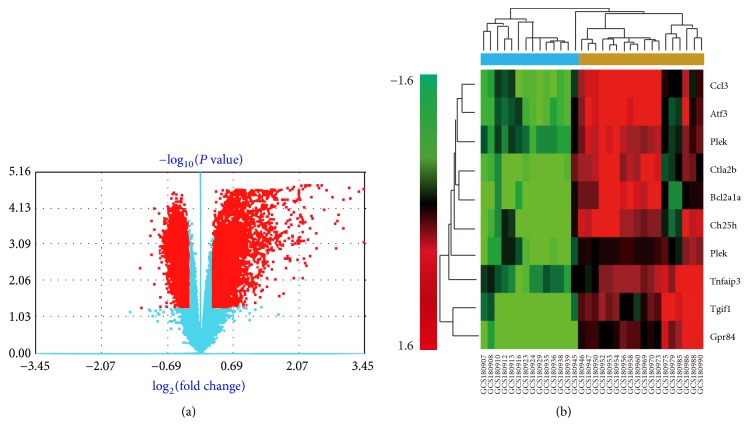
Heatmap of gene expression differences by gene coexpression network analysis. Red dot indicates a differentially expressed gene with statistical significance. Red dots on the right indicate upregulation of gene expression, whereas red dots on the left indicate downregulation of gene expression. Blue indicates that there is no statistically significant difference in gene expression. The greater the ordinate value corresponding to the point is, the greater the difference in gene expression corresponding to that point is. Similarly, the greater the absolute value of the abscissa corresponding to the point is, the greater the difference in gene expression corresponding to that point is. Note that there are 592 statistically significant DEGs. The abscissa value of Ccl3 is 3.45, which means Ccl3 is the maximum change among all DEGs (a). Hierarchical clustering dendrogram of gene expression: the horizontal axis at the bottom represents the name of samples and the vertical axis on the left side represents the degree of gene clustering. The vertical axis on the right side represents the name of genes and the horizontal axis at the top represents the degree of clustering of samples. The red color stands for upregulated while the green color stands for downregulated. The darker red indicates a stronger upregulation in expression and the darker green indicates a stronger downregulation in expression. It can be concluded that the samples can be divided into clusters generally: the control group of sham-injury and the experimental group of injury (b). Moreover, the maximum change of gene expression profile was upregulation of Ccl3 (fold change = 10.91, *P* = 2.20*E* − 05) (b).

DEGs that were significantly differentially expressed were screened out by use of significance analysis of microarrays (SAM) [35, 36] in the GCBI platform. *P* < 0.05 and fold change > 2 were used as the threshold of screening differentially expressed genes. When the number of samples becomes large, we implement a standard analysis method for screening difference genes. In fact, we use the two samples' Welch *t*-test (unequal variances) for two groups' difference analysis and use analysis of variance (ANOVA) for multiple groups (groups count no less than 3). For multiple comparison analysis, we computed the q-value to control the false discovery rate [37]. The top ten largest differences in DEGs were screened with fold change > 2 and *P* < 0.05, and the maximum change of gene expression profile was upregulated Ccl3 (Table 1). After heatmap of gene expression differences by gene coexpression network analysis, we found 592 statistically significant DEGs (Figure 2(a)), which is consistent with the known results that SCI is a polygenic disease and its pathogenic mechanism is associated with changes of gene expression. The abscissa value of Ccl3 is 3.45 (Figure 2(a)), which shows Ccl3 is the maximum change among all DEGs.

We further calculated the Pearson correlation to construct the distance between the genes and samples and implemented the hierarchical clustering based on used average method for linkage [38]. The top 10 DEGs were listed according to the size of difference and Ccl3 is the top DEGs which are upregulated. From the horizontal axis at the top, it can be concluded that the samples can be divided into clusters generally: the control group of sham-injury and the experimental group of injury (Figure 2(b)). Moreover, the maximum change of gene expression profile was upregulation of Ccl3 (fold change = 10.91, *P* = 2.20*E* − 05).

### 3.3. KEGG Pathway Enrichment Analysis from T8 Lesion Centre (MAPK Signaling Pathway Was the Most Important among the 209 Pathways)

Significantly enriched GO terms and KEGG pathways with FDR < 0.05 were screened out. The rank was according to the enrichment score, *P* value, and FDR. KEGG biological pathway enrichment analysis found that MAPK signaling pathway (enrichment score = 5.68, *P* = 3.38*E* − 74, and FDR = 8.78*E* − 72) was the most important one among the 209 pathways according to the enrichment scores (Table 2).

### 3.4. Pathway Network Analyses from T8 Lesion Centre (MAPK Signaling Pathway Was Also the Most Important Pathway)

**Figure 3:**
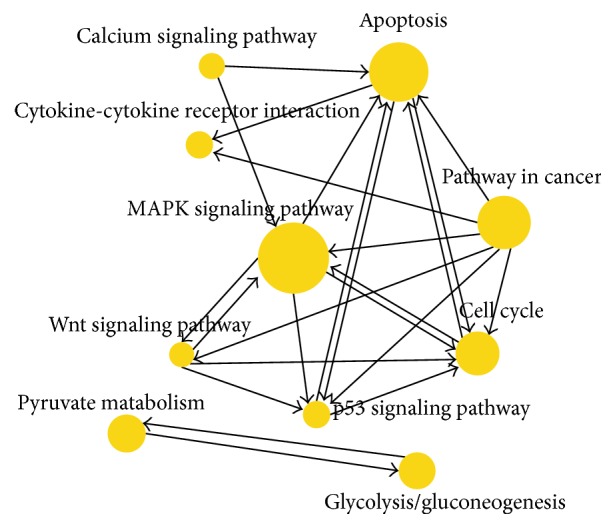
Pathway network after spinal cord injury. The more important the signaling pathway is, the larger the ball is. The importance was ranked according to the degree. MAPK signaling pathway was in the centre of the altered pathways interaction net.

The interaction in KEGG was used to construct the interaction network between pathways. The overall and systematical pathway analysis of the relationship between marked pathways can help to disclose the synergistic effect module of important pathways. In the top 10 altered pathway interaction nets with 111 nodes and 404 relationships between each other, MAPK signaling pathway was the most important one with the largest degree (outdegree = 5, indegree = 39, and degree = 44) (Table 3) and it was in the centre of the altered pathways interaction network (Figure 3).

### 3.5. Systematic Analysis of DEGs and Altered Pathway in Different Section and Time Points by SAM and KEGG

The DEG Ccl3 and MAPK signaling pathways are not necessarily in the top 10 list (Tables 4 and 5) because of the difference of data analysis. We only discussed, verified, and confirmed the correlation between DEGs Ccl3, MAPK signaling pathway, and SCI induced chronic NP because of the great diversity of genes and the huge complexity of the whole work. However, what is the most important is that systematic analysis of DEGs and altered pathway in different section and time point by SAM and KEGG suggests another method and strategy to study the target gene and pathway of nerve-related disease.

## 4. Discussion

SCI has been demonstrated to be a polygenic disease and its pathogenic mechanism is associated with changes of many genes. In this study, we have used microarray technology to identify differentially expressed genes (DEGs) and activated signaling pathways in association with SCI induced chronic NP in a mouse SCI model. We showed that Ccl3 and MAPK were the most upregulated DEG and the most activated signaling pathway, respectively. Our results are consistent with that of previous studies. It will be very interesting to further this study into SCI patients. Throughout the analysis, the factors affecting the results include sample attributes (sample source, sample size, and sample quality), treatment tools, treatment methods, and results screening. In addition, various analysis methods were used, including the screening of differentially expressed genes, KEGG pathway enrichment analyses, and pathway network analyses. All the analysis processes were performed on the GCBI platform in order to avoid the error difference resulting from running different analysis at the different platforms.

The activation of resident cells and the inflammatory cells (macrophages, neutrophils, and lymphocytes) in PNS was involved in peripheral sensitization. In the spinal dorsal horn, glial cells (microglia and astrocytes) are activated to account for central sensitization. Neuropathic pain induced by peripheral and central sensitization is mediated by some inflammatory mediators (IFMs) including chemokines and cytokines (e.g., Ccl3) [39]. After SCI, Ccl3 were induced significantly in the dorsal horns 2 days after lesion and remained at high levels with significantly higher intensities [40], while, after peripheral nerve injury, Ccl3 and their receptors (CCR2 and CCR1/CCR5, resp.) were increased [41]. In addition, there were differences in gene expression at the different stages of pain. For example, the expression of Ccl3 at the six time points was reflected in the ranking of top 10 DEGs (Table 5). Because of the existence of ongoing pain and evoked pain following SCI, here the neuropathic pain we discussed is defined as the evoked pain following SCI. Moreover, we did not perform animal experiments to confirm the relationship between the protein function and SCI-NP, and we did not exclude the false positive microarray results, which result from insufficient conditions.

Ccl3, the ligand of CCR1 [42] and CCR5 [43, 44], was upregulated after SCI and elicits chronic inflammation, resulting in NP [45, 46]. Peripheral Ccl3 [47, 48] and Ccl3 in the spinal cord [49, 50] can produce pain behaviors through the activation of chemokine receptors in the dorsal root ganglia (DRG). Ccl3 was found to be upregulated in activated Schwann cells and infiltrating macrophages close to the injured nerves and found to participate in the development of neuropathic pain through its dominant receptors CCR1 and CCR5, which are also located in Schwann cells and macrophages [39].

CCR1 were found to be induced in the early phase (first 7 days after SCI), while in the late time course (42 days after SCI) elevated chemokine levels were only found after severe SCI [42]. CCR5 was involved in the development of other inflammatory diseases through macrophage activation [51–53], which was located in primary afferent neurons or secondary neurons of the spinal dorsal horn [47, 54]. Ccl3 and its receptor, CCR5, are upregulated in the spinal cord after injury by using qRT-PCR analysis [43, 44].

Microglia and astrocytes constitutively express CCR1 and CCR5 [55, 56]. It has been shown that microglia proliferate robustly after SCI and were essential to induce NP sensitization [57, 58]. Furthermore, minocycline, a microglial inhibitor, was reported to prevent, delay, or relieve NP [59, 60]. On the contrary, microglial activation is sufficient to induce pain sensitization [61]. Microglia are referred to as a main source of IFMs in the CNS [62, 63], which plays a crucial role in neuropathic pain development [64].

MAPK signaling pathway and chemokine signaling pathway are involved in SCI, which play a very important role in SCI induced NP [65]. MAPK family includes three major members: p38, extracellular signal regulated kinase (ERK), and c-Jun N-terminal kinase (JNK), regulating different signaling pathways. MAPKs are activated by phosphorylation and transduce a broad range of extracellular stimuli by both transcriptional and nontranscriptional regulation, leading to different intracellular responses. Asiaticoside attenuates SCI induced NP through anti-inflammatory effects and inhibition of the p38-MAPK mechanism [66]. Intrathecal injection of the anti-inflammatory cytokine alleviated SCI induced inflammation, suppressing the SCI induced activation of p38-MAPK [67]. Moreover, CCR5 is one receptor of Ccl3, knockout of CCR5 suppressed SCI induced neuropathic pain [67]. Inhibition of p38 MAPK signaling pathway can alleviate neuropathic pain [68].

p38 MAPK is activated by upstream kinase MKK3/MMK6, whose activation in spinal cord microglia was reported after SCI model [69]. p38*α* and p38*β* are two major p38 isoforms among the four isoforms: *α*, *β*, *γ*, and *δ* in the mature nervous system [70]. p38*β* appears to be expressed in spinal cord microglia, and the knockdown of p38*β* but not p38*α* prevents acute pain sensitization [71]. p38 is involved in the maintenance of neuropathic pain, and its inhibitor can attenuate and reverse NP symptoms [57].

Activation of cytokine receptors (CCR1 and CCR5) results in p38 MAPK activation in spinal cord microglia. p38 activation results in increased expression, through the transcription factor NF-*κ*B or other transcription factors (e.g., ATF-2), of secreted inflammatory mediators/growth factors (e.g., cytokines and BDNF) or of genes encoding membrane receptors. In addition, p38 also induces release of PGE2 and IL-1*β* via rapid posttranslational regulation. Upon release, these mediators will sensitize nociceptive dorsal horn neurons via presynaptic and postsynaptic mechanisms, leading to persistent pain hypersensitivity [57].

Furthermore, exosomes and other extracellular vesicles are emerging as a novel form of information exchange within the nervous system, and exosomes can play both neuroprotective and neurotoxic roles [72]. Exosomes are released by neurons in a way depending on synaptic activity, and these exosomes can be retaken by other neurons, suggesting a novel way for interneuronal communication [73]. Exosomes derived from heat-stressed tumor cells (HS-TEX) which contain chemokines, such as CCL2, CCL3, CCL4, CCL5, and CCL20, could chemoattract and activate dendritic cells (DC) and T cells more potently [74]. Schwann cells-derived exosomes enhance axonal regeneration and increase neuronal survival after prodegenerative stimulation [75]. The cotransplantation of Schwann cells and OECs reduced number of astrocytes, microglia and macrophage infiltration, and the expression of chemokines (CCL2 and CCL3) at the injured site, which provide a better immune environment for SCI repair [76].

Ccl3 and its receptors, CCR5 and CCR1, are upregulated after SCI, and knockout of Ccl3 as well as inhibition of p38 MAPK signaling pathway can alleviate neuropathic pain [67]. Thus, Ccl3 antagonists may be potential new drugs for the treatment of neuropathic pain.

## 5. Conclusions

In this study, the maximum change of gene expression profile Ccl3 (fold change = 10.91, *P* = 2.20*E* − 05) was identified among the altered 529 DEGs after SCI with threshold of *P* < 0.05 and fold change > 2. Furthermore, KEGG analysis found that 209 pathways with significance were identified, among which the most important was the MAPK signaling pathway according to the enrichment score (enrichment score = 5.68, *P* = 3.38*E* − 74, and Fold Discovery Rate (FDR) = 8.78*E* − 72). According to previous study, in SCI induced chronic NP, exosomes in the peripheral blood would contain Ccl3, which was derived from Schwann cells. The exosomes could cross blood-spinal cord barrier and combine with Ccl3's receptor, CCR5, which accounts for the chronic neuropathic pain syndromes. Ccl3 and its receptor, CCR5, are also upregulated after SCI, and knockout of Ccl3 as well as inhibition of p38 MAPK signaling pathway can alleviate neuropathic pain. Since the exosomes with Ccl3 can be easily and efficiently detected in peripheral blood, Ccl3 may serve as a potentially prognostic and predictive target for the diagnosis and treatment of SCI induced chronic NP in clinical applications. What is the most, the systematic analysis of DEGs and altered pathway in different section and time point by SAM and KEGG suggests another method and strategy to study the target gene and pathway of nerve-related disease.

## Figures and Tables

**Table 1:** Top 10 significantly enriched up- and downregulated DEGs. The *P* values associated with each term are calculated by the Fisher Exact Test which represents the “degree of enrichment.” *Q*-value is the correction for multiple comparison by Benjamini and Hochberg [33]. The rank was according to the difference determined by *d* Score, fold change, and *P* value. The top ten largest differences in DEGs were screen with fold change > 2 and *P* < 0.05, and the maximum change of gene expression profile was upregulation of Ccl3.

DEGs	Accession number	Signal of control	Signal of experimental	*d* Score	Fold change	*P* value	*q*-value	Expression change	Rank
*Ccl3*	*NM_011337*	*5.37 *	*8.81 *	*10.04 *	*10.91 *	*2.20E − 05*	*0*	*Up*	*1*
Atf3	NM_007498	6.12	9.18	10.10	8.37	2.20*E* − 05	0	Up	2
Plek	NM_019549	6.67	9.20	8.10	5.77	2.20*E* − 05	0	Up	3
Ctla2b	NM_001145801	5.45	7.90	8.08	5.45	2.20*E* − 05	0	Up	4
Bcl2a1a	NM_007534	7.23	9.59	8.37	5.14	2.20*E* − 05	0	Up	5
Ch25h	NM_009890	6.82	9.13	8.27	4.95	2.20*E* − 05	0	Up	6
Plek	NM_019549	5.87	8.12	7.58	4.76	2.20*E* − 05	0	Up	7
Tnfaip3	NM_001166402	5.75	7.45	8.12	3.24	2.20*E* − 05	0	Up	8
Tgif1	NM_001164074	6.05	7.59	8.52	2.91	2.20*E* − 05	0	Up	9
Gpr84	NM_030720	6.31	7.83	7.92	2.86	2.20*E* − 05	0	Up	10

**Table 2:** Top 10 GO terms and KEGG pathways enrichment results of DEGs. Significantly enriched GO terms and KEGG pathways with FDR < 0.05 were screened out. KEGG biological pathway enrichment analysis found that MAPK signaling pathway (enrichment score = 5.68, *P* = 3.38*E* − 74, and FDR = 8.78*E* − 72) was the most important one among the 209 pathways according to the enrichment score.

Pathway ID	Pathway name	Enrichment score	*P* value	FDR	Rank
*4010*	*MAPK signaling pathway*	*5.68 *	*3.38E − 74*	*8.78E − 72*	*1*
1100	Metabolic pathways	2.73	3.22*E* − 65	4.19*E* − 63	2
4151	PI3K-Akt signaling pathway	4.31	8.74*E* − 57	7.57*E* − 55	3
5200	Pathways in cancer	4.36	2.57*E* − 53	1.67*E* − 51	4
4380	Osteoclast differentiation	6.77	2.20*E* − 52	1.14*E* − 50	5
5166	HTLV-I infection	4.54	6.12*E* − 52	2.65*E* − 50	6
4810	Regulation of actin cytoskeleton	5.06	3.43*E* − 49	1.28*E* − 47	7
4062	Chemokine signaling pathway	5.26	6.63*E* − 49	2.15*E* − 47	8
4510	Focal adhesion	5.11	2.31*E* − 47	6.68*E* − 46	9
5205	Proteoglycans in cancer	4.73	3.22*E* − 45	8.36*E* − 44	10

**Table 3:** The top 10 altered pathways of network analyses. The outdegree and indegree represent, respectively, the number of upstream and downstream signal pathways. The degree represents the sum of the outdegree and indegree. In the top 10 altered pathway interaction nets with 111 nodes and 404 relationships between each other, MAPK signaling pathway was the most important one with the largest degree (outdegree = 5, indegree = 39, and degree = 44).

Pathway ID	Pathway name	Outdegree	Indegree	Degree	Rank
*4010*	*MAPK signaling pathway*	*5*	*39*	*44*	*1*
4210	Apoptosis	3	29	32	2
5200	Pathways in cancer	28	0	28	3
4110	Cell cycle	3	20	23	4
10	Glycolysis/gluconeogenesis	5	15	20	5
4020	Calcium signaling pathway	5	14	19	6
4115	p53 signaling pathway	2	17	19	7
4310	Wnt signaling pathway	8	9	17	8
4060	Cytokine-cytokine receptor interaction	0	16	16	9
620	Pyruvate metabolism	7	8	15	10

**Table 4:** Top 10 DEGs and pathways between sham-injury and injury in lesion centre at different time points. At different time points, top 10 DEGs and pathways between sham-injury and injury in lesion centre are showed, respectively.

Time point	Top 10 DEGs between sham-injury and injury in lesion centre	Top 10 pathways between sham-injury and injury in lesion centre
0.5 h	Npas4, Gm2083, Socs3, Socs3, Fosb, Ccl3, II6, Cyr61, Ptgs2, Myh1	Pathways in cancer, MAPK signaling pathway, Transcriptional misregulation in cancer, focal adhesion, proteoglycans in cancer, PI3K-Akt signaling pathway, hippo signaling pathway, HTLV-I infection, regulation of actin cytoskeleton, metabolic pathways

4 h	Ucn2, Gm2083, Atf3, Hspa1b, Hspa1b, Ccl3, C330006P03Rik, Hspa1a, Hspa1b, Egr3	Metabolic pathways, MAPK signaling pathway, pathways in cancer, PI3K-Akt signaling pathway, HTLV-I infection, focal adhesion, proteoglycans in cancer, osteoclast differentiation, transcriptional misregulation in cancer, olfactory transduction

24 h	Gm2083, Socs3, Chi3l3, Adam8, Gp49a, Hmox1, Serpine1, Tgm1, A130040M12Rik, Tnc	Metabolic pathways, MAPK signaling pathway, pathways in cancer, HTLV-I infection, PI3K-Akt signaling pathway, focal adhesion, protein processing in endoplasmic reticulum, regulation of actin cytoskeleton, Epstein-Barr virus infection, proteoglycans in cancer

3 d	Gpnmb, Cd36, Abca1, Cd5l, Cd36, Ccnb1, Thbs1, Rrm2, Rrm2, Sprr1a	Metabolic pathways, HTLV-I infection, pathways in cancer, focal adhesion, regulation of actin cytoskeleton, PI3K-Akt signaling pathway, proteoglycans in cancer, MAPK signaling pathway, lysosome, osteoclast differentiation

7 d	Gpnmb, Gp49a, Cd36, Cd36, Ms4a7, Cd5l, C3ar1, Clec7a, Cd68, Atp6v0d2	Focal adhesion, PI3K-Akt signaling pathway, metabolic pathways, pathways in cancer, proteoglycans in cancer, MAPK signaling pathway, regulation of actin cytoskeleton, osteoclast differentiation, HTLV-I infection, tuberculosis

28 d	Gpnmb, Clec7a, Cst7, Gp49a, Lgals3, Cd68, C3ar1, Ms4a7, Sprr1a, Cd48	Metabolic pathways, MAPK signaling pathway, pathways in cancer, HTLV-I infection, focal adhesion, proteoglycans in cancer, PI3K-Akt signaling pathway, regulation of actin cytoskeleton, chemokine signaling pathway, phagosome

**Table 5:** Top 10 DEGs and pathways between sham-injury and injury in different sections. By processing data from all time points in each section, top 10 DEGs and pathways between sham-injury and injury are showed, respectively.

Section	Top 10 DEGs between sham-injury and injury	Top 10 pathways between sham-injury and injury
rostral regions	Ccl3, Plek, Slc15a3, Bcl2a1a, Plek, Tlr2, Ccl4, Clec7a, Plek, Palld	Osteoclast differentiation, cytokine-cytokine receptor interaction, PI3K-Akt signaling pathway, phagosome, Chagas disease (American trypanosomiasis), leishmaniasis, toll-like receptor signaling pathway, chemokine signaling pathway, tuberculosis, transcriptional misregulation in cancer

lesion centre	Ccl3, Atf3, Plek, Ctla2b, Bcl2a1a, Ch25h, Plek, Tnfaip3, Tgif1, Gpr84	MAPK signaling pathway, metabolic pathways, PI3K-Akt signaling pathway, pathways in cancer, osteoclast differentiation, HTLV-I infection, regulation of actin cytoskeleton, chemokine signaling pathway, Focal adhesion, proteoglycans in cancer

caudal regions	Atf3, Tlr2, Irgm1, Trim30d, S1pr3, Bcl2a1a, Slc45a3, Plek, Trim30a, Zfp36l1	Tuberculosis, phagosome, *Staphylococcus aureus* infection, leishmaniasis, osteoclast differentiation, antigen processing and presentation, herpes simplex infection, Fc gamma R-mediated phagocytosis, viral myocarditis, Toll-like receptor signaling pathway
